# Functional brain network connectivity patterns associated with midlife metabolic syndrome and cognitive vulnerabilities

**DOI:** 10.3389/fnagi.2026.1893842

**Published:** 2026-09-09

**Authors:** Isabelle Gallagher, Makenna B. McGill, David M. Schnyer, Andreana P. Haley

**Affiliations:** 1 Department of Psychology, The University of Texas at Austin, Austin, TX, United States; 2 Department of Psychology, University of Rhode Island, Kingston, RI, United States

**Keywords:** cognitive aging, default mode network, functional connectivity, metabolic syndrome, resting-state fMRI

## Abstract

**Objective:**

Metabolic syndrome (MetS) may amplify neural vulnerability via vascular and inflammatory pathways. Existing literature suggests there are alterations to functional brain network organization in adults with MetS, though the specific patterns and their relation to cognitive vulnerabilities warrant further investigation, particularly in a midlife sample.

**Method:**

Cognitively unimpaired, community-dwelling adults (*N* = 223; ages 40–65; MetS = 73) were included. Resting-state fMRI data were preprocessed with fMRIPrep and analyzed using the Schaefer 400-parcel atlas. Functional connectivity (FC) metrics included within- and between-network connectivity, system segregation, modularity, and global efficiency. Cognitive performance was measured via neuropsychological tests for memory and executive functioning. Generalized linear models (adjusted for age, sex, framewise displacement) were used to examine group differences in FC metrics and associations between FC metrics and cognitive performance.

**Results:**

MetS participants showed reduced within-network connectivity in the default mode network (DMN; adjusted *p* = 0.038, partial *η*^2^ = 0.035). Follow-up component-level analyses indicated abdominal obesity and low HDL cholesterol were associated with reduced connectivity in DMN (abdominal obesity: adjusted *p* = 0.027, partial *η*^2^ = 0.029; cholesterol: adjusted *p* = 0.002, partial *η*^2^ = 0.058). Reduced DMN connectivity was associated with poorer verbal fluency (adjusted *p* = 0.026, partial *R*^2^ = 0.037). Exploratory analyses identified age and sex as factors that interacted with MetS group to influence FC metrics (*p*’s < 0.050, partial *η*^2^’s = 0.020–0.030).

**Conclusion:**

Midlife MetS was associated with modest reductions in within-network DMN connectivity, with abdominal obesity and low HDL cholesterol as the primary contributing MetS components. Reduced DMN connectivity was associated with poorer verbal fluency performance. These findings suggest that subtle differences in higher-order functional brain network organization associated with metabolic syndrome can be detected during midlife and are related to cognitive functioning, highlighting the importance of prevention and intervention of cardiometabolic risk in midlife to preserve network integrity and cognitive health.

## 1 Introduction

Middle age is a pivotal period during which cardiometabolic risk factors begin to accumulate, increasing vulnerability to later-life cognitive decline and dementia (Whitmer et al., 2005; Yaffe et al., 2020). Metabolic syndrome (MetS) is a clustering of cardiometabolic risk factors affecting more than two in five U.S. adults (Liang et al., 2023), defined by the presence of three or more of the following conditions: abdominal obesity, hypertension, elevated triglycerides, reduced high-density lipoprotein cholesterol, and elevated fasting glucose concentrations (Alberti et al., 2009). MetS increases risk for age-related neurodegenerative diseases, including Alzheimer’s disease and related dementias (Beeri and Gu, 2024; Qureshi et al., 2024).

The impact of cardiometabolic risk on brain aging may be mediated through vascular and inflammatory pathways. The 2024 Lancet Commission on dementia prevention highlights hypertension, obesity, diabetes, and dyslipidemia as modifiable risk factors that contribute to cerebrovascular dysfunction, chronic low-grade inflammation, and impaired metabolic regulation (Livingston et al., 2024). These processes can compromise cerebral perfusion (Wolters et al., 2017; Livingston et al., 2020), blood–brain barrier integrity (Van Dyken and Lacoste, 2018), and cell signaling (Gerardo et al., 2025), providing biologically plausible mechanisms through which individual cardiometabolic risk components may influence large-scale brain network organization. In addition to vascular dysfunction, metabolic abnormalities common in MetS, particularly insulin resistance, have been associated with reduced cerebral blood flow and altered regional cerebral glucose metabolism (Baker et al., 2011; Birdsill et al., 2013), supporting the hypothesis that disrupted neurovascular and metabolic pathways contribute to alterations in large-scale brain network organization. Resting-state functional connectivity is derived from fluctuations in blood oxygen level-dependent (BOLD) signal, an indirect measure of neural activity that depends on cerebral hemodynamic responses. Age-related alterations in cerebral perfusion, glucose metabolism, and functional brain networks occur in parallel, suggesting that metabolic and hemodynamic dysfunction many contribute to changes in large-scale brain network organization (Deery et al., 2023). Functional connectivity, a measure of coordinated neural activity across distributed brain systems, has been shown to change across the lifespan in the context of both normative aging and neurodegenerative disease and may be particularly impacted by vascular (Wirth et al., 2022) and inflammatory mechanisms (Nusslock et al., 2019).

Several studies in older or later-middle-aged adults with MetS or elevated cardiometabolic risk report altered functional connectivity, consistently highlighting differences in the default mode network (DMN), despite using varied neuroimaging analysis approaches. Later-middle-aged adults with MetS show reduced DMN connectivity to other brain regions (Rashid et al., 2019) as well as hyperconnectivity across core regions and loss of modularity (Rashid et al., 2021). One study using an aggregate index of cardiometabolic and vascular risk, irrespective of specific diagnoses, found reduced global and regional connectivity, particularly in insular, medial frontal, medial parietal, and superior temporal regions, as well as between core networks including the DMN, cingulo-opercular, and dorsal attention networks (Rashid et al., 2023). These studies demonstrate that MetS is associated with functional connectivity differences in higher-order brain regions in later-middle-aged adults; however, none assessed cognition, leaving the behavioral impact of these connectivity differences unclear. Few studies examining individual cardiometabolic risk factors have including cognitive measures as outcomes. In older adults with type 2 diabetes, studies reported reduced DMN connectivity, with Cui et al. (2015) further showing that decreased posterior cingulate connectivity correlated with poorer visual delayed recall and executive performance (Musen et al., 2012; Cui et al., 2015). In older adults with obesity, higher BMI was associated with reduced posterior DMN connectivity, which was linked to poorer executive functioning (Beyer et al., 2017). Collectively, these findings indicate that cardiometabolic risk is associated with altered global and regional functional connectivity in populations that skew to older adults. It remains unclear whether similar MetS-related connectivity patterns and cognitive vulnerabilities emerge earlier in midlife.

Earlier midlife represents a critical period when early identification of individuals at risk for cognitive decline may be particularly informative, given the functional and structural topological changes occurring in the brain between the 30s and 60s (Deery et al., 2023; Mousley et al., 2025). According to the Scaffolding Theory of Aging and Cognition, cardiometabolic burden in midlife may contribute to weakened neural resources (Reuter-Lorenz and Park, 2014), increasing susceptibility to cognitive decline and neurodegenerative disease. This framework highlights the importance of integrating comprehensive health and cognitive assessments with functional brain network connectivity analyses to detect subtle neural vulnerabilities. Disruptions in the higher-order brain networks, comprising prefrontal and connected affective and control regions that support internally directed cognition and executive regulation (Menon, 2023), have been linked to memory and executive function declines (Deery et al., 2023).

Building on our previous work demonstrating that midlife MetS was associated with structural brain aging (Gallagher et al., 2025), the present study extends these findings by incorporating resting-state fMRI to examine whether MetS is also associated with differences in functional brain network organization and whether these functional connectivity differences relate to cognitive performance. The present study therefore (1) examined resting-state functional connectivity differences associated with midlife MetS and individual cardiometabolic components, and (2) evaluated whether these functional connectivity differences were associated with cognitive performance. We hypothesized that adults with MetS would demonstrate exacerbated age-related differences in functional connectivity (e.g., decreased within-network connectivity, decreased network segregation and modularity, decreased global efficiency, and increased between-network connectivity) and that these differences would be associated with individual cardiometabolic components, particularly abdominal obesity. We further hypothesized that differences in functional connectivity patterns would be associated with poorer cognitive performance. Lastly, exploratory analyses examined whether associations between functional connectivity and MetS varied by age and sex.

## 2 Methods

### 2.1 Participants and study design

This study design includes secondary use of data from a previously published study examining metabolic and structural brain health (Gallagher et al., 2025). The current study extends prior work by incorporating resting-state fMRI to examine functional brain network organization in relation to cardiometabolic health and cognitive functioning. Participants were cognitively unimpaired, community-dwelling adults aged 40–65 without a diagnosis of a neurological or psychiatric disorder. Detailed inclusion/exclusion criteria, recruitment, and baseline assessments are reported in Gallagher et al. (2025). In short, participants were excluded for the following reasons: age above 65; incomplete demographic, cardiometabolic, cognitive, or neuroimaging data; a score less than 24 on the Mini-Mental State Examination; and cognitive scores greater or less than 2.5 standard deviations from the sample mean on cognitive measures. This resulted in a sample of 230 participants in our prior publication; in the present resting-state functional MRI study, an additional 7 participants were removed during quality control of mean framewise displacement. The final analytic sample therefore included 223 participants (184 from Study 1 and 39 from Study 2, as previously described in Gallagher et al., 2025). All participants provided written informed consent before enrolling in the study. Study procedures were completed in accordance with the Helsinki Declaration and approved by the Institutional Review Board at the University of Texas at Austin.

### 2.2 Cardiometabolic health assessment and metabolic syndrome identification

Cardiometabolic assessments were conducted as previously described in detail (Foret et al., 2020; Gallagher et al., 2025). Briefly, fasting blood measures included glucose, triglycerides, high-density lipoprotein (HDL) cholesterol, low-density lipoprotein (LDL) cholesterol, total cholesterol, and blood pressure and anthropometrics were collected following standardized protocols. Medical and medication history, including post-menopausal status (yes/no/other; defined as 12 consecutive months without a menstrual cycle), was obtained through self-report.

Participants were classified as having MetS if they met three or more of the five metabolic syndrome criteria defined by the Joint Interim Statement (Alberti et al., 2009): (1) abdominal obesity (waist circumference ≥102 cm for men, ≥88 cm for women); (2) elevated triglycerides (≥150 mg/dL), or use of triglyceride lowering medication; (3) low HDL cholesterol (<40 mg/dL for men, <50 mg/dL), or use of cholesterol medication; (4) elevated blood pressure (systolic ≥130 mmHg or diastolic ≥85 mmHg), or use of antihypertensive medication; and (5) elevated fasting glucose (≥100 mg/dL), or use of glucose-lowering medication. Medication use was incorporated into the MetS classification according to the Joint Interim Statement criteria. Participants meeting fewer than three criteria (0–2 components) were classified as not having MetS. The presence of each MetS component and the total number of components were also recorded for each participant.

### 2.3 Neuropsychological assessment

Participants completed a battery of neuropsychological tests, as detailed in prior publications (Foret et al., 2021). Participants also completed measures of estimated intellectual functioning (WASI-II FSIQ-2 or WTAR). The present analysis included measures across cognitive domains that have been shown to be associated with cardiometabolic health risk in adults (Foret et al., 2021; Lamar et al., 2021): (1) verbal learning [California Verbal Learning Test—Second Edition (CVLT-II) Trials 1–5 Total], (2) delayed recall (CVLT-II Long Delay Free Recall), (3) recognition memory (CVLT-II Recognition Discriminability) (Delis et al., 2000), (4) working memory (Digit Span Backward) (Wechsler, 2008); (5) verbal fluency [Controlled Oral Word Association (COWA)] (Benton et al., 1983); and (6) executive functioning [Trail Making Test (TMT) Part B time minus Part A time; reverse-scored] (Reitan, 1955). Cognitive scores were demographically adjusted for age, sex, and years of education and subsequently *z*-scored.

### 2.4 MRI data acquisition

Magnetic resonance imaging (MRI) was conducted on a Siemens 3T Skyra with a 32-channel head coil at the Biomedical Imaging Center (RRID: SCR_021898), a core facility within the Center for Biomedical Research Support at the University of Texas at Austin. A high-resolution T1-weighted structural image was acquired using a magnetization-prepared rapid gradient echo (MPRAGE) sequence [256 × 256 matrix; flip angle = 7°; field of view (FOV) = 24 × 24 cm^2^; slice thickness = 1 mm; no interslice gap]. Resting-state functional MRI (rs-fMRI) data were collected during a 6-min scan using a gradient-echo echo-planar imaging sequence [repetition time (TR) = 3,000 ms; echo time (TE) = 30 ms; FOV = 24 × 24 cm^2^; 64 × 64 matrix; 42 axial slices; slice thickness = 3 mm; interslice gap = 0.3 mm]. During the rs-fMRI acquisition, participants were instructed to keep their eyes open and fixate on a central crosshair.

### 2.5 Resting-state fMRI processing and functional connectivity analysis

#### 2.5.1 Preprocessing

Resting-state fMRI data were preprocessed using fMRIPrep (v25.2.3) (Esteban et al., 2019). Anatomical preprocessing included skull stripping and bias-field correction (Tustison et al., 2010), cortical surface reconstruction using FreeSurfer (Dale et al., 1999), brain mask estimation (Klein et al., 2017), nonlinear normalization to MNI152 standard space (Fonov et al., 2009; Avants et al., 2011), and tissue segmentation of cerebrospinal fluid (CSF), white matter (WM), and gray matter (GM) (Zhang et al., 2001).

Functional preprocessing included slice-timing correction (Cox, 1996) and head motion correction (Jenkinson et al., 2002), followed by co-registration to the corresponding T1-weighted image (Greve and Fischl, 2009). Motion-correction transforms, BOLD-to-T1-weighted transforms, and T1-weighted-to-MNI warps were concatenated and applied in a single step using antsApplyTransforms (ANTs v2.1.0) with Lanczos interpolation. Physiological noise regressors were derived using the CompCor method (Behzadi et al., 2007). Framewise displacement (FD) was calculated for each run using the Nipype implementation (Power et al., 2014).

#### 2.5.2 Denoising

Denoising was performed using confound regressors generated by fMRIPrep, following a standard regression strategy (Ciric et al., 2017). Regressors included: (1) six rigid-body motion parameters (three translations and three rotations); (2) mean WM and CSF signals; (3) global signal and (4) temporal high-pass filtering (cutoff = 0.008 Hz) (Power et al., 2012). No additional low-pass filter was applied, consistent with our denoising strategy, which combined nuisance regression with high-pass filtering while retaining a broader temporal frequency range (Lindquist et al., 2019). Global signal regression was included to reduce widespread physiological and motion-related sources of variance to improve to the functional connectivity estimates for characterizing large-scale functional brain network organization (Murphy and Fox, 2017).

#### 2.5.3 Quality control

Quality assurance procedures included both automated and manual assessments. Automated quality metrics were obtained using fMRIPrep (Esteban et al., 2019) and MRIQC (Esteban et al., 2017). All functional and anatomical images were visually inspected for artifacts and gross anatomical abnormalities. No participants were identified with poor-quality data to be excluded. Participant imaging data were retained only if mean framewise displacement was ≤0.5 mm (*n* = 7 subjects removed).

#### 2.5.4 Functional connectivity and brain network metrics

**Figure 1:**
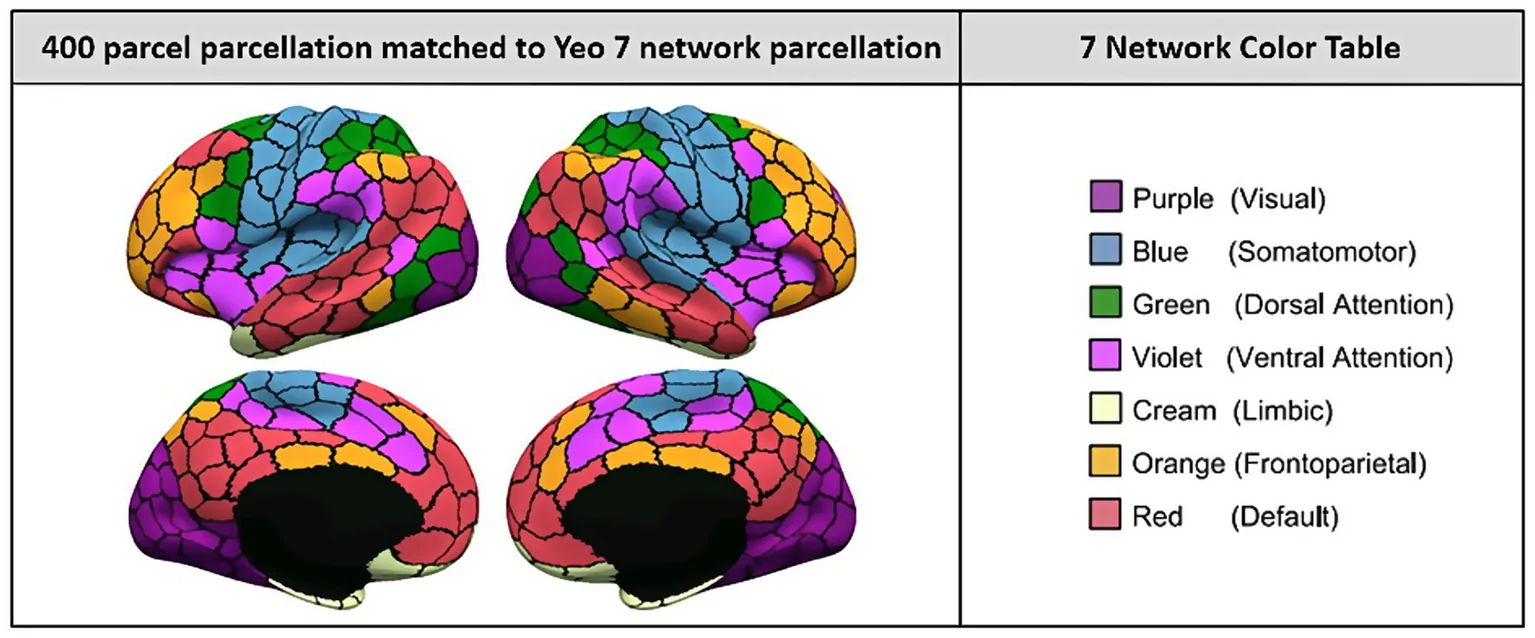
7-network parcellation. A 400-parcel cortical parcellation matched to the Yeo 7-network parcellation was used for functional connectivity analyses. Reproduced from Schaefer et al. (2018), by permission of Oxford University Press; figure obtained from the authors’ GitHub repository. (https://github.com/ThomasYeoLab/CBIG/tree/master/stable_projects/brain_parcellation/Schaefer2018_LocalGlobal)

Whole-brain functional connectivity matrices were treated as weighted, undirected graphs. Functional connectivity matrices were derived using the Schaefer 400 parcel atlas with Yeo 7-network assignments (Schaefer et al., 2018) (see Figure 1). Within-network connectivity was defined as the mean Fisher *z*-transformed correlation coefficients among regions within the same functional network and computed using the upper triangle of each within-network submatrix. Between-network connectivity was defined as the mean Fisher *z*-transformed correlation coefficients across pairwise connections between regions belonging to different functional networks. System segregation was estimated at the whole-brain level using the predefined network assignments. System segregation was computed in *R* as (*W* − *B*)/*W*, where *W* represents mean correlation between nodes within same network and *B* represents mean correlation between nodes belonging to different networks (Chan et al., 2014). Applying the method described by Chan et al. (2014), negative edge weights and diagonal elements were set to zero prior to computation. Additional brain network measures were computed using the Brain Connectivity Toolbox (Rubinov and Sporns, 2010), implemented in Python (bctpy v0.6.1). Prior to computation of graph-theoretic metrics, negative edge weights were set to zero and diagonal elements were removed, consistent with recommendations for weighted graph metrics that are defined for nonnegative edge weights (Rubinov and Sporns, 2011). Modularity was estimated using the Louvain community detection algorithm for weighted, undirected graphs implemented in the Brain Connectivity Toolbox (modularity_louvain_und). The modularity quality index (*q*) reflects the extent to which the network can be partitioned into communities with dense within-community connectivity and sparse between-community connectivity. Network integration was quantified using global efficiency, computed using the Brain Connectivity Toolbox (efficiency_wei). For weighted graphs, global efficiency was calculated as the average inverse shortest path length between all pairs of nodes, with distances derived from inverse edge weights, and reflects the efficiency of parallel information transfer across the whole-brain network.

### 2.6 Statistical analyses

All analyses were conducted using R version 4.3.0. Statistical significance was set at *p* < 0.05. Differences in sample and clinical characteristics across the MetS groups and individual components were examined using independent samples *t*-tests and chi-square tests of independence. Analysis of covariance (ANCOVA) models were used to examine differences in FC metrics based on MetS group status and individual metabolic components. Primary models were adjusted for age, sex, and mean framewise displacement, with effect sizes reported as partial *η*^2^. To account for multiple comparisons across the 7 Yeo networks, raw *p*-values were adjusted using the Benjamini–Hochberg false discovery rate (FDR) method at *q* < 0.05. FDR correction was applied separately for the MetS group status analysis across (i) within-network connectivity across the 7 Yeo networks, (ii) between-network connectivity across all 21 network-pair connections, and (iii) global graph-theoretic metrics (mean within-network connectivity, mean between-network connectivity, system segregation, modularity, and global efficiency; 5 tests). A sensitivity analysis additionally adjusted the significant MetS-related functional connectivity finding for predicted age difference (PAD), a measure of structural brain aging previously examined in this sample (Gallagher et al., 2025). For networks identified as differing between the MetS groups, follow-up ANCOVAs examined associations between individual MetS components and FC metrics, adjusting for age, sex, and mean framewise displacement. FDR correction was applied across the five MetS component tests. Multiple linear regression was then performed across the entire sample (*N* = 223) to examine associations between these FC metrics and sample specific cognitive *z*-scores. Models were adjusted for age, sex, and mean framewise displacement, with effect sizes reported as partial *R*^2^. FDR correction was applied across the six cognitive tests.

Exploratory moderation analyses were conducted using ANCOVA models to examine whether associations between MetS group and global functional connectivity metrics varied as a function of age and sex. Separate models were conducted for age and sex interactions. Age moderation models included main effects of MetS group, age, sex, and mean framewise displacement (FD), as well as the MetS × age interaction term. Sex moderation models included main effects of MetS group, sex, age, and mean FD, as well as the MetS × sex interaction term. General linear models were used to examine main and interaction effects, and effect sizes were reported as partial *η*^2^. Significant interactions were further probed using simple effects and simple slope analyses. FDR correction was applied separately within each interaction family (MetS × age and MetS × sex).

The present study represents a secondary analysis of an existing cohort, as such, an *a priori* sample size calculation was not performed. A sensitivity power analysis indicated that the available sample (*N* = 223; MetS = 73, non-MetS = 150) provided 80% power (*α* = 0.05) to detect effects of approximately partial *η*^2^/*R*^2^ = 0.034, indicating adequate power for small-to-moderate effects.

## 3 Results

### 3.1 Sample and clinical characteristics

Demographic and clinical characteristics are presented in Table 1, and are similar to those reported in prior work (Gallagher et al., 2025). Seventy-three participants met the clinical criteria for MetS as established by Alberti et al. (2009). There were no significant differences across MetS groups on demographic variables (*p*’s > 0.050). There were significant differences in MetS component variables, such that the MetS group displayed a greater presence of each individual MetS component (*p*’s < 0.001), as expected. There were significant group differences for some cognitive test scores, such that the MetS group displayed poorer performance on measures of verbal learning and memory (*p*’s < 0.050). There was not a significant difference in estimated IQ, which demonstrated average estimated intellectual functioning for both groups. There was a significant difference in mean framewise displacement between MetS and no MetS (*p* = 0.024).

**Table 1:** Sample and clinical characteristics.

Selected demographic	Clinical characteristics	MetS (*n* = 73)	No MetS (*n* = 150)	Test statistic (*t* or *X*^2^)	*p*
Age [mean (SD)]		50.29 (6.87)	50.68 (6.96)	0.40	0.691
Sex [*n* (*n*%)]	Female	36 (49.32)	94 (62.67)	3.07	0.080
	Male	37 (50.68)	56 (37.33)		
Race/ethnicity [*n* (*n*%)]	Asian	2 (2.74)	7 (4.67)	5.34	0.376
	Black	3 (4.11)	11 (7.33)		
	Hispanic	17 (23.29)	25 (16.67)		
	Multiracial	1 (1.37)	0 (0.00)		
	Other	4 (5.48)	5 (3.33)		
	White	45 (61.64)	102 (68.00)		
	N/A	1 (1.37)	0 (0.00)		
Years of education [mean (SD)]		16.01 (2.75)	16.55 (2.38)	1.44	0.153
MMSE total score [mean (SD)]		28.60 (1.45)	28.73 (1.55)	0.62	0.539
Estimated IQ [mean (SD)]					
WASI-II FSIQ-2^a^		110.52 (16.26)	114.20 (13.85)	1.57	0.119
WTAR^b^		107.00 (10.55)	111.66 (6.66)	0.86	0.447
Metabolic syndrome components					
MetS components [mean (SD)]		3.68 (0.74)	0.92 (0.77)	−25.73	<0.001
Abdominal obesity [*n* (*n*%)]	Absent	7 (9.59)	99 (66.00)	60.41	<0.001
	Present	66 (90.41)	51 (34.00)		
Elevated triglycerides [*n* (*n*%)]	Absent	18 (24.66)	135 (90.00)	94.34	<0.001
	Present	55 (75.34)	15 (10.00)		
Low HDL cholesterol [*n* (*n*%)]	Absent	16 (21.92)	133 (88.67)	95.68	<0.001
	Present	57 (78.08)	17 (11.33)		
Elevated blood pressure [*n* (*n*%)]	Absent	29 (39.73)	114 (76.00)	26.53	<0.001
	Present	44 (60.27)	36 (24.00)		
Elevated glucose [*n* (*n*%)]	Absent	26 (35.62)	131 (87.33)	60.57	<0.001
	Present	47 (64.38)	19 (12.67)		
Cognitive test performance^c^					
CVLT-II Trials 1–5 [mean (SD)]		−0.27 (1.00)	0.14 (0.98)	2.93	0.004
CVLT-II LDFR [mean (SD)]		−0.37 (0.94)	0.17 (0.99)	3.96	<0.001
CVLT-II Recognition Discriminability [mean (SD)]		−0.23 (0.94)	0.09 (1.02)	2.34	0.020
COWA [mean (SD)]		−0.10 (1.04)	0.07 (0.99)	1.15	0.252
Digit Span Backward [mean (SD)]		0.00 (1.03)	0.03 (0.99)	0.21	0.831
TMT B-A [mean (SD)]		0.04 (1.07)	−0.03 (0.99)	−0.44	0.664
Resting-state fMRI					
Mean framewise displacement [mean (SD)]		0.22 (0.12)	0.18 (0.09)	−2.29	0.024

COWA, Controlled Oral Word Association; CVLT-II, California Verbal Learning Test—Second Edition; fMRI, functional magnetic resonance imaging; FSIQ-2, Full Scale Intelligence Quotient-2; HDL, high-density lipoprotein; IQ, intelligence quotient; LDFR, Long Delay Free Recall; MetS, metabolic syndrome; MMSE, Mini-Mental Status Examination; SD, standard deviation; TMT, Trail Making Test (Part B minus Part A; reverse-scored); WASI-II, Wechsler Abbreviated Scale of Intelligence—2nd Edition; WTAR, Weschler Test of Adult Reading.

a Study 1 participants only (*n* = 184).

b Study 2 participants only (*n* = 39).

c Sample-specific *z*-scores adjusted for age, sex, and years of education.

Demographic characteristics (i.e., age, sex) for individual MetS components are presented in Table 2. Participants with the low HDL cholesterol component were significantly younger than those without the component, and participants with the blood pressure component were significantly older than those without the component (*p*’s < 0.050). Regarding sex differences, there were higher proportions of males with the triglycerides, low HDL cholesterol, and glucose components relative to females (*p*’s < 0.010). There was a higher proportion of females with the abdominal obesity component relative to males (*p* < 0.001).

**Table 2:** Sample characteristics for individual metabolic syndrome components.

MetS component		MetS component present	MetS component absent	Test statistic (*t* or *X*^2^)	*p*
Abdominal obesity					
Age [mean (SD)]		49.82 (6.72)	51.36 (7.08)	1.66	0.098
Sex [*n* (*n*%)]	Female	81 (69.23)	49 (46.23)	11.18	<0.001
	Male	36 (30.77)	57 (53.77)		
Elevated triglycerides					
Age [mean (SD)]		51.54 (6.95)	50.10 (6.88)	−1.44	0.151
Sex [*n* (*n*%)]	Female	30 (42.86)	100 (65.36)	9.10	0.003
	Male	40 (57.14)	53 (34.64)		
Low HDL cholesterol					
Age [mean (SD)]		48.92 (6.74)	51.36 (6.89)	2.53	0.012
Sex [*n* (*n*%)]	Female	33 (44.59)	97 (65.10)	7.73	0.005
	Male	41 (55.41)	52 (34.90)		
Elevated blood pressure					
Age [mean (SD)]		51.77 (6.88)	49.87 (6.87)	−1.99	0.049
Sex [*n* (*n*%)]	Female	49 (61.25)	81 (56.64)	0.28	0.598
	Male	31 (38.75)	62 (43.36)		
Elevated glucose					
Age [mean (SD)]		50.73 (6.75)	50.48 (7.01)	−0.25	0.804
Sex [*n* (*n*%)]	Female	28 (42.42)	102 (64.97)	8.81	0.003
	Male	38 (57.58)	55 (35.03)		

MetS, metabolic syndrome; HDL, high-density lipoprotein; SD, standard deviation.

Self-reported menopausal status was descriptively examined across MetS groups for female participants (Table 3). In the MetS group, 52.78% of participants reported being postmenopausal, compared to 44.68% in the no MetS group. No significant group differences in menopausal status were observed (*p* = 0.390).

**Table 3:** Self-reported menopause status in female sample.

Menopause status [n (n%)]		MetS (*n* = 36)	No MetS (*n* = 94)	Test statistic (*X*^2^)	*p*
Menopause status [*n* (*n*%)]	Post-menopause	19 (52.78)	42 (44.68)	1.87	0.39
	Not post-menopause	16 (44.44)	43 (45.74)		
	Other	0 (0.00)	4 (4.26)		
	N/A	1 (2.77)	5 (5.32)		

MetS, metabolic syndrome.

### 3.2 MetS participants displayed reduced within-network connectivity

**Figure 2:**
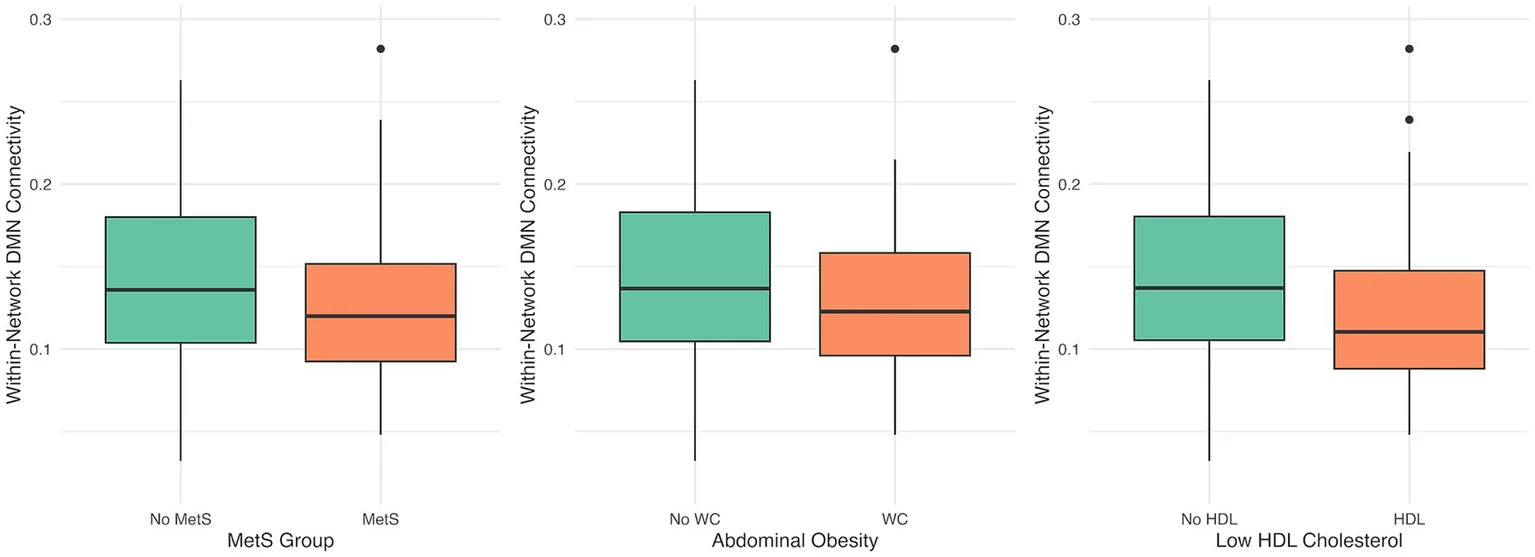
Differences in functional connectivity by MetS group and individual MetS components. Group differences in functional connectivity are shown. Individuals with MetS demonstrated lower FC within the default mode network. Among MetS components, abdominal obesity (elevated waist circumference) and low high-density lipoprotein were each associated with reduced FC within the default mode networks. FC, functional connectivity; HDL, high-density lipoprotein; MetS, metabolic syndrome; WC, waist circumference.

ANCOVAs adjusted for age, sex, and mean framewise displacement revealed that, relative to participants without MetS, those with MetS displayed reduced within-network connectivity in the DMN [*F*(1, 218) = 7.88, adjusted *p* = 0.038, partial *η*^2^ = 0.035, Figure 2], A similar pattern was observed in the salience/ventral attention network [SVAN; *F*(1, 218) = 5.95, *p* = 0.016, partial *η*^2^ = 0.027], although this association did not remain significant after FDR correction (adjusted *p* = 0.054). No other significant differences in within-, between-, or global FC metrics were identified between the MetS groups after FDR correction (adjusted *p*’s > 0.050, Supplementary Table 1). In a sensitivity analysis, the association between MetS and reduced within-network DMN connectivity remained significant after additionally adjusting for PAD [*F*(1, 217) = 7.91, *p* = 0.005], while PAD was not independently associated with DMN connectivity [*F*(1, 217) = 1.63, *p* = 0.203].

### 3.3 Abdominal obesity and low HDL cholesterol components were associated with reduced within-network connectivity

For the DMN, which showed significant group differences, ANCOVAs adjusted for age, sex, and mean framewise displacement were conducted to examine differences in within-network DMN connectivity for individual MetS components. Participants meeting criteria for the abdominal obesity component displayed reduced connectivity within the DMN [*F*(1, 218) = 6.60, adjusted *p* = 0.027, partial *η*^2^ = 0.029], relative to those without the abdominal obesity component. Similarly, participants meeting criteria for the low HDL cholesterol component displayed reduced connectivity within the DMN [*F*(1, 218) = 13.40, adjusted *p* = 0.002, partial *η*^2^ = 0.058], relative to those without the low HDL cholesterol component (see Figure 2). There were no significant differences in within-network DMN connectivity for the other MetS components (i.e., triglycerides, blood pressure, glucose; adjusted *p*’s > 0.050) (see Supplementary Table 2).

### 3.4 Reduced within-network DMN connectivity was associated with poorer verbal fluency

The DMN was the only network that differed significantly between MetS groups and therefore was selected for follow-up cognitive analyses. Across the full sample (*N* = 223), linear regressions adjusted for age, sex, and mean framewise displacement were used to examine associations between within-network DMN connectivity and sample-specific cognitive *z*-scores. Reduced connectivity within the DMN was significantly associated with poorer verbal fluency [*t*(218) = 2.88, adjusted *p* = 0.026, partial *R*^2^ = 0.037]. There were no significant associations between within-network connectivity and other cognitive *z*-scores (adjusted *p*’s > 0.050) (see Supplementary Table 3).

### 3.5 Exploratory analyses

**Figure 3:**
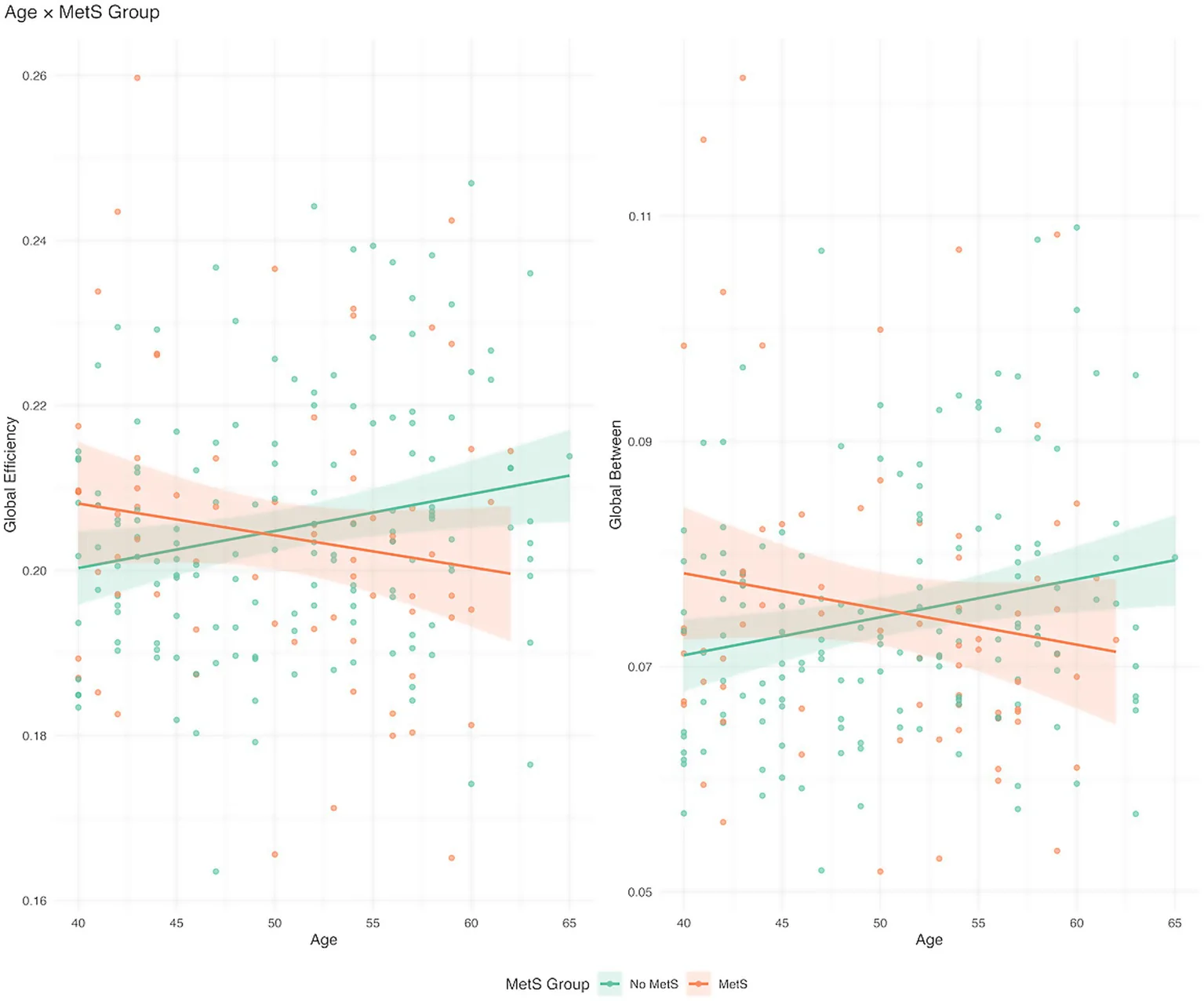
Interaction effects between MetS group and age on functional connectivity. Exploratory interactions between metabolic syndrome and age are shown. MetS × age significant interaction was observed for global efficiency and global between-network connectivity. Among individuals with MetS, older age was associated with lower connectivity, whereas younger individuals with MetS showed higher connectivity. In contrast, individuals without MetS demonstrated relatively preserved connectivity across age. MetS, metabolic syndrome.

Exploratory ANCOVAs adjusted for sex and mean framewise displacement revealed significant MetS group × age interactions on global efficiency [*F*(1, 217) = 6.64, unadjusted *p* = 0.011, adjusted *p* = 0.043, partial *η*^2^ = 0.030] and global between-network connectivity [*F*(1, 217) = 5.762, unadjusted *p* = 0.017, adjusted *p* = 0.043, partial *η*^2^ = 0.026]. Follow-up slope analyses indicated that the association between age and connectivity metrics differed by MetS group, such that participants without MetS demonstrated positive age-related associations, whereas participants with MetS demonstrated negative age-related associations across metrics (Figure 3).

Exploratory ANCOVAs adjusted for age and mean framewise displacement revealed a MetS group × sex interaction on global between-network connectivity [*F*(1, 217) = 4.45, unadjusted *p* = 0.036, partial *η*^2^ = 0.020; Supplementary Figure 1]. Follow-up simple effects analyses indicated that males with MetS demonstrated lower between-network connectivity relative to females with MetS (unadjusted *p* = 0.029), whereas no sex differences were observed among participants without MetS (unadjusted *p* = 0.581). Within-sex comparisons indicated no significant differences between MetS groups among females or males, although males with MetS showed a trend toward lower connectivity relative to males without MetS. A MetS group × sex interaction was also observed for system segregation [*F*(1, 217) = 4.39, unadjusted *p* = 0.037, partial *η*^2^ = 0.020; Supplementary Figure 1]. Follow-up analyses indicated that males without MetS demonstrated lower system segregation relative to females without MetS (unadjusted *p* = 0.020), whereas no sex differences were observed among participants with MetS. Within-sex comparisons between MetS groups were not significant for either males or females. However, neither MetS × sex interaction survived Benjamini–Hochberg FDR correction (adjusted *p*’s = 0.093; Supplementary Table 4). No other MetS × age or MetS × sex interactions were observed for global functional connectivity metrics (*p*’s > 0.050).

## 4 Discussion

The present study provides evidence of small-to-moderate differences in functional brain network organization associated with midlife MetS. Participants with MetS exhibited reduced connectivity within the DMN compared with individuals without MetS, with abdominal obesity and low HDL cholesterol identified as key MetS components. The observed group- and component-level differences were small in magnitude, suggesting subtle alterations in functional network organization rather than large-scale disruptions. Reduced DMN connectivity was also associated with poorer verbal fluency performance.

Our primary finding of reduced within-network DMN connectivity in MetS participants is consistent with prior research in older adults showing that MetS and related cardiometabolic burden are associated with reduced functional connectivity in higher-order brain networks (Musen et al., 2012; Cui et al., 2015; Beyer et al., 2017; Rashid et al., 2019, 2023), and extend those observations to a midlife sample, suggesting that subtle network-level differences may be detected earlier in the life course. Our findings differ from those reported by Rashid et al. (2021), who observed widespread increases in functional connectivity in adults with MetS. This may reflect sample and/or methodological differences (e.g., broader age range, seed-based approach; see Supplementary Table 5 for details) and highlights the importance of future studies using harmonized analytic pipelines and longitudinal designs to better determine how functional network organization changes across the progression of metabolic syndrome. Despite these methodological differences, evidence across studies suggest that cardiometabolic risk is associated with alterations in functional connectivity of higher-order brain networks, particularly the DMN.

In the present analysis, midlife MetS-related differences were observed in the DMN which has been linked to cognitive aging and vascular health. The DMN has been consistently implicated in studies of normative aging, Alzheimer’s disease pathology, and in predicting Alzheimer’s disease and related dementia risk (Greicius and Menon, 2004; Hedden et al., 2009; Brier et al., 2014; Ereira et al., 2024). Similarly, alterations in ventral attention network connectivity have been reported in individuals with elevated cardiometabolic risk (Sadler et al., 2021) and are reduced in the context of greater white matter hyperintensities associated with cardiovascular disease (Zhu et al., 2023). Our findings, along with prior research, suggest that functional connectivity metrics in higher-order networks may capture subtle neural vulnerabilities associated with midlife MetS, even in cognitively unimpaired adults.

At the component level, abdominal obesity and low HDL cholesterol emerged as the only components individually associated with reduced within-network connectivity in the DMN. This finding aligns with previous work that identified reduced DMN connectivity in older adults with elevated body mass index (Beyer et al., 2017). Central adiposity, as reflected in waist circumference, has been linked to systemic inflammation, insulin resistance, and cerebrovascular alterations that can disrupt neural signaling and network organization (De A Boleti et al., 2023). Similarly, elevated cholesterol contributes to vascular dysfunction, atherosclerosis, and reduced cerebral perfusion (Meyer et al., 1987; Herzog et al., 2025), and has been shown to be associated with age-related decoupling (i.e., decreased network connectivity with age) in early to midlife adults (Spielberg et al., 2017). While underlying biological mechanisms were not directly examined in the present study, our findings are in line with recent evidence emphasizing vascular and inflammatory mechanisms as central pathways linking cardiometabolic risk to cognitive decline, particularly how both mechanisms can occur simultaneously and place increased demands on systemic health (Jin et al., 2023; Tian et al., 2023). Other MetS components, including triglycerides, blood pressure, and glucose, did not show independent associations with DMN network connectivity in the current study, suggesting that certain cardiometabolic factors may contribute differently to functional brain network organization. In contrast, our previous work in this sample demonstrated that MetS was associated with structural brain aging, with triglycerides emerging as the primary metabolic component associated with brain age acceleration (Gallagher et al., 2025), further supporting the notion that individual metabolic components may exert partially distinct effects on structural and/or functional brain measures. The divergence across MetS components raises the possibility that individual cardiometabolic risk factors may influence brain aging through multiple pathways prior to overt cognitive impairment.

We also sought to examine whether differences in functional connectivity as observed in MetS participants were associated with cognitive performance. Reduced DMN connectivity was associated with poorer phonemic verbal fluency, which is thought to be a task not only of language abilities but also executive functioning (Aita et al., 2019). Numerous studies have identified a link between reductions in DMN connectivity and executive functioning (Raichle, 2015), including a few studies examining cardiometabolic risk (Cui et al., 2015; Beyer et al., 2017). Interestingly, in our sample the MetS groups did not significantly differ on measures of executive functioning (including verbal fluency), which was consistent with our prior work in this sample that identified an association between the MetS group and patterns of poorer memory, but not poorer executive functioning (Gallagher et al., 2025). Taken together, our two studies suggest that structural and functional brain differences are detectable in midlife MetS alongside subtle but distinct cognitive correlates, even as cognitive vulnerabilities remain minimal. Longitudinal study of midlife adults may aid in examining whether associations between network-level alterations and cognitive performance become more pronounced in adults with MetS as they age.

Exploratory analyses indicated interactions between MetS group and demographic factors (age, sex) on functional connectivity. Age-related significant interactions suggest that older participants with MetS exhibited reduced global efficiency and between-network connectivity, supporting that age-associated reductions in network integration observed in normal aging (Deery et al., 2023) may be more pronounced in the context of cardiometabolic burden across the life course.

Additionally, exploratory sex-related interaction analyses identified patterns that did not survive correction for multiple comparisons. Specifically, males with MetS exhibited reduced global between-network connectivity than females with MetS, whereas males without MetS demonstrated reduced system segregation than females without MetS. Although preliminary, these findings are of interest given that sex is an important contributor to the physiology and clinical expression of MetS (Kuk and Ardern, 2010), and cardiometabolic risk trajectories diverge across midlife, particularly across the menopausal transition (El Khoudary et al., 2020; Liang et al., 2023). In the present sample, overall MetS prevalence did not differ by sex, though individual MetS components showed distinct sex-related patterns, with central adiposity (waist circumference) more prevalent in females, and dyslipidemia-related components (low high-density lipoprotein cholesterol) more prevalent in males. These differences in cardiometabolic health profiles may contribute to heterogeneity in functional brain network organization. More broadly, MetS components have been linked to distinct neuroinflammatory and vascular pathways that support brain network organization. Central adiposity has been linked to chronic low-grade inflammation and endocrine dysregulation (Kawai et al., 2021), whereas dyslipidemia is more strongly associated with vascular dysfunction and atherosclerotic burden (Du and Qin, 2023). Although the mechanisms underlying these exploratory findings remain speculative, these observations highlight demographic heterogeneity as an important area for future research. Replication in larger, adequately powered studies designed to examine demographic heterogeneity in MetS-associated functional brain organization is needed to determine whether these patterns reflect stable neural vulnerability or dynamic, potentially modifiable changes in brain network integrity.

Strengths of this study include the use of comprehensive neuropsychological and cardiovascular assessments, combined with advanced neuroimaging and analytic approaches to characterize large-scale brain network organization in a midlife sample, a developmental period that remains underrepresented in the literature. The integration of both global and network-specific measures of functional connectivity provides a nuanced understanding of how MetS may relate to brain organization.

Several limitations should also be considered. Firstly, the cross-sectional design of the study precludes causal inference, and there is potential for cohort effects or selection bias. Secondly, the effect sizes observed were generally small (partial *η*^2^ ≈ 0.020–0.058). While sensitivity analyses indicated that the study was sufficiently powered to detect effects as small as partial *η*^2^ = 0.03, null findings should be interpreted cautiously. Thirdly, the rs-fMRI acquisition was 6 min in duration. While this acquisition is sufficient for estimating group-level resting-state functional connectivity, longer acquisitions can improve the reliability of functional connectivity estimates, particularly at the individual-participant level (Birn et al., 2013; Han et al., 2024). Accordingly, the present findings should be interpreted as group-level associations within the constraints of the acquisition protocol and replicated in future studies using longer acquisitions where feasible. Fourthly, medication use was incorporated into MetS classification according to standard consensus criteria. However, we were not sufficiently powered to disentangle measured cardiometabolic burden from medication characteristics (e.g., class, dose, treatment duration) across individual MetS components. Future studies should examine raw cardiometabolic measures alongside detailed medication characterization to clarify whether functional network differences are associated with untreated physiological burden, treated disease, medication-specific effects, or treatment intensity. Lastly, future longitudinal studies are needed to determine the trajectory of network changes and their predictive value for cognitive decline and neurodegenerative risk. Continuing to incorporate granular assessments of individual MetS components, as well as sex- and age-related biological factors, will be critical for understanding the mechanisms underlying these associations.

In summary, midlife MetS was associated with modest differences in within-network DMN connectivity, with abdominal obesity and low HDL cholesterol influencing these patterns. Reduced DMN connectivity, a higher-order functional brain network, was also associated with poorer verbal fluency performance. These findings underscore the importance of monitoring and addressing cardiometabolic risk in midlife to support functional network integrity and cognitive health.

## Data Availability

The raw data supporting the conclusions of this article will be made available by the authors, without undue reservation.

## Supplementary material

SUPPLEMENTARY FIGURE 1 Interaction effects between MetS group and sex on functional connectivity. Exploratory interactions between metabolic syndrome and sex are shown. MetS × sex interaction was observed for global between-network connectivity and system segregation. Men with MetS exhibited reduced between connectivity relative to females with MetS. Men without MetS showed reduced system segregation relative to females without MetS. Neither MetS × sex interaction survived FDR correction. MetS, metabolic syndrome.
