# Supplementary material for: Functional brain network connectivity patterns associated with midlife metabolic syndrome and cognitive vulnerabilities
Source: Front Aging Neurosci. 2026 Sep 9;18:1893842. doi: 10.3389/fnagi.2026.1893842 (PMC13598075; doi:10.3389/fnagi.2026.1893842)
Supplement: Supplementary file 1 [file Table_1.DOCX]

**Supplemental Table 1: Differences in functional connectivity metrics by metabolic syndrome group status**

|  |  | **Test statistic (F)** | | **p _unadjusted_** | **p _adjusted_** | **partial η^2^** |
| --- | --- | --- | --- | --- | --- | --- |
| **Within network metrics** | | |  |  |  |  |
| Within Visual | | | 0.744 | 0.3894 | 0.6814 | 0.003 |
| Within Somatomotor | | | 1.337 | 0.2488 | 0.5806 | 0.006 |
| Within DAN | | | <0.001 | 0.9912 | 0.9912 | <0.001 |
| Within SVAN | | | 5.953 | 0.0155 | 0.0542 | 0.027 |
| Within Limbic | | | 0.164 | 0.6857 | 0.8000 | <0.001 |
| Within FPN | | | 0.455 | 0.5006 | 0.7008 | 0.002 |
| Within DMN | | | 7.885 | 0.0054 | 0.0381 | 0.035 |
| **Between network metrics** | | |  |  |  |  |
| Visual - Somatomotor | | | 0.090 | 0.7651 | 0.9844 | <0.001 |
| Visual - DAN | | | 0.140 | 0.7087 | 0.9844 | <0.001 |
| Visual - SVAN | | | <0.001 | 0.9844 | 0.9844 | <0.001 |
| Visual - Limbic | | | 0.074 | 0.7852 | 0.9844 | <0.001 |
| Visual - FPN | | | 1.039 | 0.3093 | 0.9844 | 0.005 |
| Visual - DMN | | | 1.294 | 0.2566 | 0.9844 | 0.006 |
| Somatomotor - DAN | | | 0.003 | 0.9587 | 0.9844 | <0.001 |
| Somatomotor - SVAN | | | 1.638 | 0.2019 | 0.9844 | 0.007 |
| Somatomotor - Limbic | | | 1.724 | 0.1905 | 0.9844 | 0.008 |
| Somatomotor - FPN | | | 0.045 | 0.8321 | 0.9844 | <0.001 |
| Somatomotor - DMN | | | 0.010 | 0.9217 | 0.9844 | <0.001 |
| DAN - SVAN | | | 0.253 | 0.6152 | 0.9844 | 0.001 |
| DAN - Limbic | | | 1.953 | 0.1637 | 0.9844 | 0.009 |
| DAN - FPN | | | 0.250 | 0.6173 | 0.9844 | 0.001 |
| DAN - DMN | | | 0.433 | 0.5114 | 0.9844 | 0.002 |
| SVAN - Limbic | | | 0.006 | 0.9383 | 0.9844 | <0.001 |
| SVAN - FPN | | | 0.597 | 0.4405 | 0.9844 | 0.003 |
| SVAN - DMN | | | 1.787 | 0.1826 | 0.9844 | 0.008 |
| Limbic - FPN | | | 0.570 | 0.4509 | 0.9844 | 0.003 |
| Limbic -DMN | | | 0.709 | 0.4008 | 0.9844 | 0.003 |
| FPN - DMN | | | 0.279 | 0.5981 | 0.9844 | 0.001 |
| **Global metrics** | | |  |  |  |  |
| Within (mean) | | | 3.982 | 0.0472 | 0.2361 | 0.018 |
| Between (mean) | | | 0.061 | 0.8053 | 0.8053 | <0.001 |
| System segregation | | | 2.444 | 0.1194 | 0.2986 | 0.011 |
| Modularity | | | 0.124 | 0.7256 | 0.8053 | <0.001 |
| Global efficiency | | | 0.168 | 0.6819 | 0.8053 | <0.001 |

DAN=dorsal attention network; DMN=default mode network; FPN=frontoparietal network; MetS=metabolic syndrome; SVAN=salience/ventral attention network. Analysis of covariance (ANCOVA) models adjusted for age, sex, and mean framewise displacement. Effect sizes are reported as partial η^2^ and rounded to the nearest third decimal. To account for multiple comparisons across networks, raw p-values were adjusted using the Benjamini-Hochberg false discovery rate (FDR) method. Raw and adjusted p-values are rounded to the nearest fourth decimal. FDR correction was applied separately for within, between, and global functional connectivity metrics.

**Supplemental Table 2:** **Differences in within default mode network functional connectivity by metabolic syndrome components**

|  |  | | **Test statistic (F)** | **p _unadjusted_** | **p _adjusted_** | **partial η^2^** |
| --- | --- | --- | --- | --- | --- | --- |
| **Abdominal obesity** | |  | |  |  |  |
| Within DMN | | 6.596 | | 0.0109 | 0.0272 | 0.029 |
| **Elevated triglycerides** | |  | |  |  |  |
| Within DMN | | 0.101 | | 0.7512 | 0.7512 | <0.001 |
| **Low HDL cholesterol** | |  | |  |  |  |
| Within DMN | | 13.397 | | 0.0003 | 0.0016 | 0.058 |
| **Elevated blood pressure** | |  | |  |  |  |
| Within DMN | | 2.270 | | 0.1333 | 0.1667 | 0.010 |
| **Elevated glucose** | |  | |  |  |  |
| Within DMN | | 3.582 | | 0.0597 | 0.0996 | 0.016 |

DMN=default mode network; HDL=; MetS=metabolic syndrome. Analysis of covariance (ANCOVA) models adjusted for age, sex, and mean framewise displacement. Effect sizes are reported as partial η^2^ and rounded to the nearest third decimal. To account for multiple comparisons across components, raw p-values were adjusted using the Benjamini-Hochberg false discovery rate. Raw and adjusted p-values are rounded to the fourth decimal.

**Supplemental Table 3:** **Associations between default mode within network functional connectivity and cognitive performance**

|  |  | | **Test statistic (t)** | **p _unadjusted_** | **p _adjusted_** | **partial R^2^** |
| --- | --- | --- | --- | --- | --- | --- |
| **Within DMN** | |  | |  |  |  |
| CVLT-II Trials 1-5 | | -0.177 | | 0.8596 | 0.8596 | <0.001 |
| CVLT-II LDFR | | -0.670 | | 0.5037 | 0.6751 | 0.002 |
| CVLT-II RD | | -0.583 | | 0.5606 | 0.6751 | 0.002 |
| COWA | | 2.881 | | 0.0044 | 0.0262 | 0.037 |
| Digit Span Backward | | 2.100 | | 0.0369 | 0.1107 | 0.020 |
| TMT B-A | | 0.580 | | 0.5626 | 0.6751 | 0.002 |

COWA=Controlled Oral Word Association; CVLT-II=California Verbal Learning Test – Second Edition; DMN=default mode network; LDFR=long delay free recall; MetS=metabolic syndrome; RD=recognition discriminability; TMT=Trail Making Test (Part B minus Part A; reverse-scored). Multiple linear regression models adjusted for age, sex, and mean framewise displacement. Effect sizes are reported as partial R^2^ and rounded to the third decimal. Cognitive scores were demographically adjusted for age, sex, and years of education prior to being z-scored. To account for multiple comparisons across cognitive tests, raw p-values were adjusted using the Benjamini-Hochberg false discovery rate method. Raw and adjusted p-values are rounded to the fourth decimal.

**Supplemental** **Table 4:** **Exploratory age × MetS and sex × MetS interaction effects on global network metrics**

|  | **Test statistic (F)** | | **p _unadjusted_** | | **p  _adjusted_** | | **partial η^2^** |  |
| --- | --- | --- | --- | --- | --- | --- | --- | --- |
| **age x MetS** | |  | |  | |  |  | |
| Within (mean) | | 3.534 | | 0.0615 | | 0.1024 | 0.016 | |
| Between (mean) | | 5.762 | | 0.0172 | | 0.0431 | 0.026 | |
| System segregation | | 0.157 | | 0.693 | | 0.694 | 0.001 | |
| Global efficiency | | 6.639 | | 0.0106 | | 0.0431 | 0.030 | |
| Modularity | | 3.034 | | 0.0830 | | 0.1037 | 0.014 | |
| **sex x MetS** | |  | |  | |  |  | |
| Within (mean) | | 0.071 | | 0.7905 | | 0.7905 | <0.001 | |
| Between (mean) | | 4.446 | | 0.0361 | | 0.0934 | 0.020 | |
| System segregation | | 4.388 | | 0.0374 | | 0.0934 | 0.020 | |
| Global efficiency | | 2.566 | | 0.1107 | | 0.1844 | 0.012 | |
| Modularity | | 0.477 | | 0.4906 | | 0.6132 | 0.002 | |

MetS=metabolic syndrome; FDR=false discovery rate. General linear models adjusted for age, sex, and mean framewise displacement. Effect sizes are reported as partial R^2^ and rounded to the third decimal. Raw p-values and Benjamini–Hochberg false discovery rate (FDR)-adjusted p-values are shown and rounded to the nearest fourth decimal. FDR correction was applied separately within each interaction family (MetS × age and MetS × sex).

**Supplemental Table 5: Methodological comparison between the present study and Rashid et al. (2021) examining functional connectivity in metabolic syndrome.**

|  | **Present Study** | **Rashid et al. (2021)** |
| --- | --- | --- |
| Sample size | N=223 (No MetS=150; MetS=73) | N=78 (No MetS=51; MetS=27) |
| Sample age | Range: 40-65; groups age-matched  Mean (SD): 50.68 (6.96) [No MetS]; 50.29 (6.87) [MetS] | Range: 30-90; MetS group significantly older  Mean (SD): 59.63 (8.60) [No MetS];  65.70 (7.87) [MetS] |
| Recruitment | Community-based midlife cohort | VA Boston clinics and community recruitment |
| MetS diagnostic criteria | Harmonized consensus criteria from Alberti et al., 2009; ≥ 3 of 5 components including medication:  (1) Abdominal obeisty (waist circumference ≥102 cm for men, ≥88 cm for women);  (2) elevated triglycerides (≥150 mg/dL), or use of triglyceride lowering medication;  (3) low HDL cholesterol (<40 mg/dL for men, <50 mg/dL), or use of cholesterol medication;  (4) elevated blood pressure (systolic ≥130 mmHg or diastolic ≥85 mmHg), or use of blood pressure medication;  (5) elevated fasting glucose (≥100 mg/dL), or use of insulin regulating medication | Classification criteria from Grundy, 2005;  ≥ 3 of the following component risk factors:  (1). elevated waist circumference ≥102/88 cm (male/female)  (2)elevated triglycerides ≥150 mg/dL or drug treatment for dyslipidemia  (3)reduced HDL-C <40/50 mg/dL (male/female) or drug treatment for dyslipidemia  (4)elevated systolic BP ≥130 mmHg or diastolic BP ≥85 mmHg or drug treatment  for hypertension,  (5) elevated fasting plasma glucose ≥100 mg/dL or drug treatment for elevated glucose or diabetes |
| MRI scanner | Siemens Skyra 3T, 32-channel head coil | Siemens Prisma Fit 3T (n = 70) and TIM Trio 3T (n = 8), 32-channel head coil |
| Resting-state acquisition | TR/TE=3000/30, FOV=24x24 cm², 64x64 matrix, 42 axial slices, slice thickness=3 mm, interslice gap=0.3 mm, 6 minutes/run) | TR/TE=4000/31, FOV=128x128 mm, voxel size=2x2x2.5 mm, 55 axial slices, 90 volumes, 6:12 minutes/run) |
| Preprocessing | fMRIPrep pipeline including motion correction, CompCor-based nuisance regression, global signal regression, censoring, and Fisher z-transformed functional connectivity matrices | FSFAST/FreeSurfer, AFNI, and FSL pipeline including motion correction, global signal regression, white matter/CSF regression, band-pass filtering, and surface-based projection |
| FC analytic approach | Schaefer 400-parcel atlas with Yeo 7-network assignment; parcel-to-parcel connectivity matrices used to compute participant-level network metrics | Yeo 7-network seeds used to generate seed-to-whole-brain voxel-wise connectivity maps |
| Primary FC outcome | Participant-level within-network connectivity and graph-theoretic metrics (system segregation, modularity, global efficiency) | Seed-based voxel-wise functional connectivity difference maps summarized as within- and between-network vertex counts |
| Graph Theory Metrics | Included (system segregation, modularity, global efficiency) | Not used |
| Statistical Approach | ANCOVA adjusted for age, sex, mean FD; Benjamini-Hochberg FDR correction | Vertex-wise GLM adjusted for age, sex, scanner; Monte Carlo cluster-wise family-wise error correction |
| Unit of Analysis | Participant-level connectivity matrices | Vertex-wise seed-based group maps |
| Main finding | Reduced within-network connectivity in the DMN; no differences in global graph metrics after FDR correction | Widespread increases in seed-based functional connectivity and reduced modular organization |
| Interpretive Implication | Subtle reductions in participant-level network integrity in cognitively unimpaired midlife adults | Widespread seed-based connectivity increases and reduced network modularity in an older, methodologically distinct cohort |
